# The evaluation of Congo red staining combined with fluorescence microscopy in the diagnosis of primary cutaneous amyloidosis

**DOI:** 10.1111/1346-8138.17562

**Published:** 2024-12-12

**Authors:** Hanqing Song, Yin Cheng, Xiuqin Wang, Xinyi Hong, Ze Guo, Hui Li, Li Li, Peiguang Wang

**Affiliations:** ^1^ Department of Dermatology The First Affiliated Hospital, Anhui Medical University Hefei Anhui China; ^2^ Institute of Dermatology Anhui Medical University Hefei Anhui China; ^3^ Key Laboratory of Dermatology (Anhui Medical University), Ministry of Education Hefei Anhui China; ^4^ Collaborative Innovation Center of Complex and Severe Skin Disease Anhui Medical University Hefei Anhui China; ^5^ Department of Pathology The First Affiliated Hospital, Anhui Medical University Hefei Anhui China

**Keywords:** amyloid, Congo red, diagnosis, fluorescence microscopy, primary cutaneous amyloidosis

## Abstract

Primary cutaneous amyloidosis (PCA) is a chronic pruritic skin disease. The apple‐green birefringence of Congo red‐stained amyloid under a polarized light microscope (CR‐PLM) remains the gold standard in the diagnosis of PCA. However, there are some limitations to this approach. In this study, eighty‐two paraffin‐embedded biopsy skin samples were collected from patients with a clinical diagnosis of PCA. The sections were respectively stained with hematoxylin–eosin (HE), crystal violet (CV), and Congo red (CR) and observed under a light microscope. CR‐stained sections were also observed under a polarized light microscope (CR‐PLM) or an ultraviolet (UV)‐emitted fluorescence microscope (CR‐UFM). Further, 35 cases clinically diagnosed with psoriasis, lichen planus, and prurigo nodularis were selected as the negative control group. The positive rate of amyloid protein detected by CR‐UFM (81.71%) was significantly higher than that detected by CR‐PLM (70.73%, *p* = 0.004), CR staining (56.10%, *p* < 0.001), CV staining (30.49%, *p* < 0.001), or HE staining (28.05%, *p* < 0.001). In the control group, 34 (97.14%) cases were negative for amyloid deposits in CR staining, CR‐PLM, and CR‐UFM sections. The relative number of positive dermal papillae observed by CR‐UFM (0.35 ± 0.27) was much more than that observed by CR‐PLM (0.15 ± 0.17, *p*<0.001), CR staining (0.12 ± 0.16, *p* < 0.001), CV staining (0.07 ± 0.12, *p* < 0.001), or HE staining (0.05 ± 0.12, *p* < 0.001). The intensity of fluorescence by CR‐UFM was significantly greater than that of the appl‐green birefringence by CR‐PLM (*p* < 0.001). Moreover, the amyloid was easily distinguished from the surrounding tissues using the CR‐UFM method. In conclusion, the CR‐UFM method was superior to CR‐PLM, CR staining, CV staining, and HE staining in diagnosing PCA.

## 1 INTRODUCTION

Primary cutaneous amyloidosis (PCA) is a group of chronic and itchy dermatoses characterized by the accumulation of amyloid in the superficial dermis without the involvement of internal organs.^1^ Quality of life is impacted to varying degrees for most patients with PCA due to the intense pruritus and unsightly appearance of the disease.^2^, ^3^ Clinically, the disease usually manifests as lichenoid papules, nodules, and hyperpigmented macules. Other symptoms also occur, such as blisters, skin atrophy, and hypopigmented spots.^4^, ^5^ The diagnosis of PCA remains challenging when physicians are unfamiliar with its clinical appearance.^6^, ^7^

Although the typical clinical features may suggest PCA, its definite diagnosis requires determination of the amyloid by skin biopsy. The amyloid appears as homogenous amorphous substances in the dermis^8^ and can easily be confused with other similar substances in hematoxylin–eosin (HE)‐, crystal violet (CV)‐, or Congo red (CR)‐stained sections under an ordinary optical microscope.^9^ Amyloid is highlighted by CR staining under a polarized light microscope (CR‐PLM)^10^ and exhibits a characteristic apple‐green birefringence. The apple‐green birefringence is considered the gold standard for confirming amyloid protein.^8^, ^11^ However, the method is not really reliable, especially in cases with few amyloid deposits.^12^, ^13^ Furthermore, the CR‐stained amyloid may exhibit multiple hues under a polarized light, and the unique apple‐green birefringence is observed only when two filters are accurately crossed.^14^, ^15^

The method of Congo red staining combined with an ultraviolet (UV)‐emitted fluorescence microscope (CR‐UFM) has been used to evaluate amyloid protein in a few studies.^16^, ^17^ The aim of this study was to investigate the value of a CR‐UFM approach in the diagnosis of PCA.

## 2 MATERIALS AND METHODS

### 2.1 Clinical data and skin samples

We collected clinical data and paraffin‐embedded biopsy skin samples from 82 patients with a clinical diagnosis of PCA between 2010 and 2023. These patients exhibited typical skin lesions of PCA.^18^ Forty (48.78%) male patients and 42 (51.22%) female patients were included. Their mean age was 43.70 ± 15.63 years (range, 18–85 years); mean age at disease onset was 36.35 ± 14.10 years (range, 10–72 years). We also collected skin samples from 35 patients clinically diagnosed with psoriasis vulgaris, lichen planus, and prurigo nodularis. These samples were used as a control group.

### 2.2 The histochemical staining of skin samples

The paraffin‐embedded skin samples were sliced into 5‐μm‐thick continuous sections. The tissue sections were then dewaxed by xylene and rehydrated with an ethanol gradient (100%, 95%, 85%, and 75% ethanol). HE and CV staining were performed on the sections according to standard protocols. Additionally, CR staining was performed using a modified method described by Highman.^19^ Each slide was evaluated and counted independently by two experienced dermatopathologists in a double‐blind fashion.

### 2.3 The evaluation of HE‐, CV‐ and CR‐stained amyloid under an ordinary light source

All stained sections were examined under an ordinary light source of a multiple‐purpose microscope (Olympus BX‐53). The amyloid was evaluated as homogeneous amorphous eosinophilic substances within the dermal papillae in the HE‐stained sections, purplish‐violet substances in the CV‐stained sections, and brick‐red substances in the CR‐stained sections.

### 2.4 The evaluation of CR‐stained amyloid protein under a polarized light device

The CR‐stained sections were examined under a cross‐polarized light accessory device of the BX‐53 multiple‐purpose microscope. The amyloid exhibits a characteristic apple‐green birefringence. The luminance intensity of birefringence was graded as no birefringence (0), weak birefringence (1, only visible at high magnification), moderate birefringence (2, easily visible at low magnification), and strong birefringence (3, strongly positive at low magnification).^20^

### 2.5 The evaluation of CR‐stained amyloid under an UV‐emitted fluorescence microscope

The CR‐stained sections were also examined under a UV‐emitted fluorescence device of the BX‐53 multiple‐purpose microscope using a UV filter set (excitation filter 340–390 nm and absorption filter 420 nm IF). The amyloid exhibits a bright purplish‐red ore‐like contour under the excitation of UV light with 340–390 nm wavelength. The luminance intensity of amyloid fluorescence was graded as no fluorescence (0), weak fluorescence (1, only visible at high magnification), moderate fluorescence (2, easily visible at low magnification), and strong fluorescence (3, strongly positive at low magnification).

### 2.6 The counting of amyloid positive areas

An independent dermal papillae containing amyloid protein is considered a positive area. We counted the number of positive dermal papillae and the total number of dermal papillae and calculated the relative number of positive dermal papillae as the ratio of the number of positive dermal papillae to the total number of dermal papillae in each section.

### 2.7 Verification of amyloid deposits by transmission electron microscope

We collected skin biopsy samples from four patients with PCA. The samples were fixed in glutaraldehyde at 4°C, rinsed four times for 1 h each in buffer, post‐fixed in 1% osmium acid at 4°C for 4 h, and rinsed three times for 10 min each in ddH20. The samples were successively stained with 2% uranium acetate for 2 h, dehydrated with gradient ethanol, infiltrated and embedded in epoxy resin (Epon812), then sliced into ultrathin sections (70–100 nm), and finally stained with lead citrate. After drying, the prepared slices were observed under the transmission electron microscopy. Electron microscope shows the amyloid is composed of long, non‐branching straight filaments with a diameter of 6.0–10.0 nm that are randomly arranged without bundle formation.

### 2.8 Evaluation of amyloid deposits under CR‐UFM in 19 cases of systemic amyloidosis

Nineteen tissue samples with systemic amyloidosis were collected from the vocal cord, thyroid, ureter, nasopharynx, eyelid, lung, parotid, urinary bladder, abdominal fat, and colon of the patients. The CR‐stained sections were observed under an ordinary light microscope, a polarized light microscope, and an UV‐emitted fluorescence microscope, respectively.

### 2.9 Statistical analysis

The data analysis was performed using the IBM SPSS software, version 27 (IBM Corp.). We used the McNemar test to compare the positive rates of five detection methods including HE staining, CV staining, CR staining, CR‐PLM, and CR‐UFM. The mean ratio of the number of positive dermal papillae to the total number of dermal papillae were compared by using the Wilcoxon rank‐sum test between CR‐UFM and the other four methods. In addition, we used the Wilcoxon rank‐sum test to compare the brightness intensities of apple‐green birefringence under CR‐PLM and fluorescence under CR‐UFM for the amyloid deposits. Significance was set at *p* < 0.05.

## 3 RESULTS

### 3.1 Histopathological findings of five methods

**FIGURE 1:**
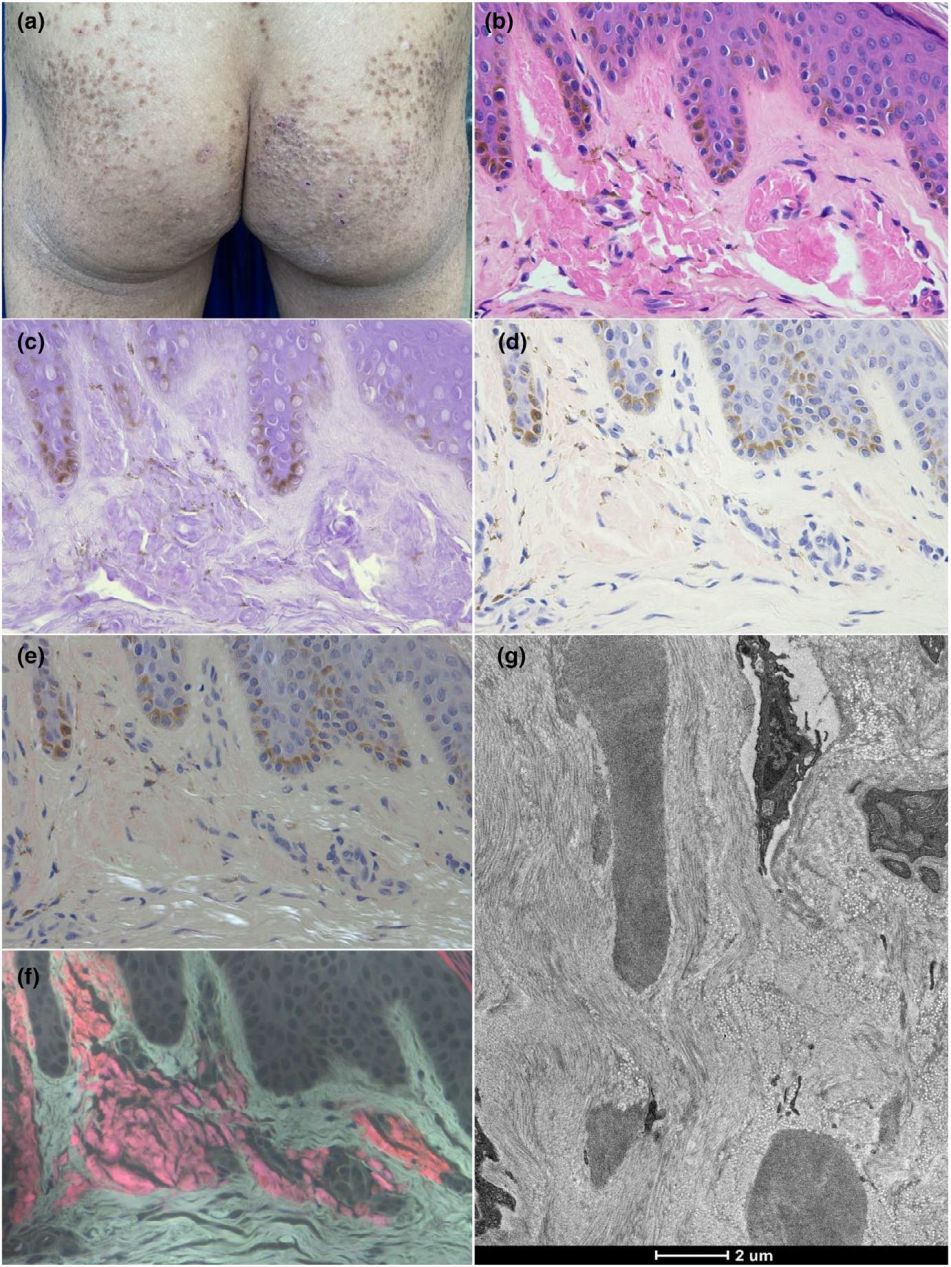
(a) Skin lesions on the buttocks. (b–g) the histopathological findings of hematoxylin–eosin, crystal violet, Congo red, Congo red under a polarized light microscope, Congo red ultraviolet‐emitted fluorescence microscope (original magnification×200), and transmission electron microscopy (original magnification×3400) from one patient with lichen amyloidosis.

**FIGURE 2:**
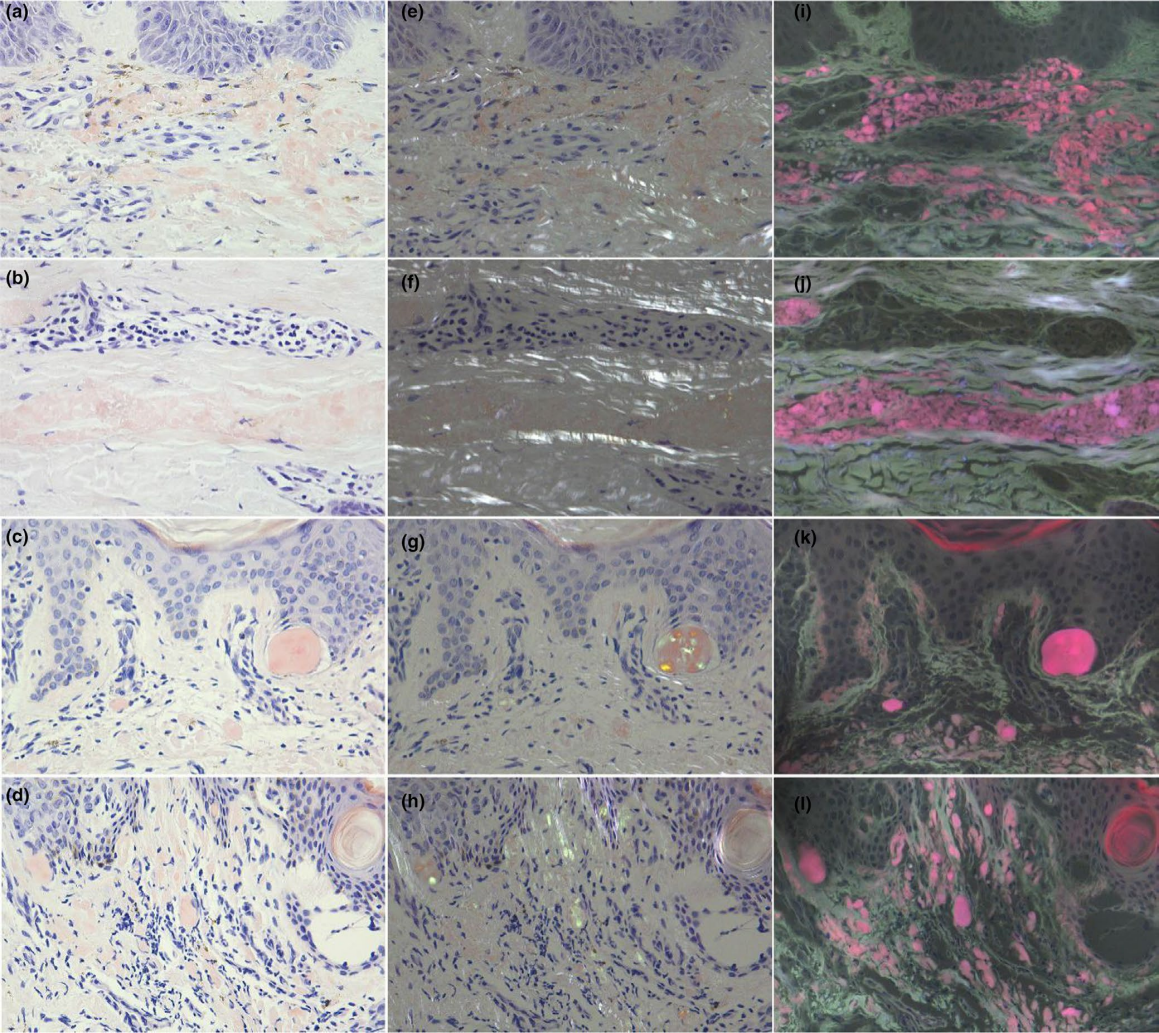
(a‐d) The histopathological findings of Congo red, (e‐h) Congo red under a polarized light microscope, and (i‐l) ultraviolet‐emitted fluorescence microscope CR‐UFM from four primary cutaneous amyloidosis patients (original magnification×200).

The positive results of five methods were showed in one patient with lichen amyloidosis, and transmission electron microscopy (TEM) confirmed the presence of amyloid in the dermal papillae (Figure 1). In addition, amyloid was observed by TEM in the other three patients with PCA. The amyloid stained with HE, CV or CR respectively showed amorphous homogeneous pink, purplish‐violet, and brick‐red clump‐like material in the papillae of dermis, which were not easily distinguishable from the surrounding dermal tissues. The CR‐stained amyloid exhibited apple‐green birefringence in the superficial dermis under a polarized light microscope. Bright purplish‐red ore‐like amyloid material was clearly observed in the dermal papillae, while the coloring of epidermal keratinocytes was gray, and the coloring of dermal tissues was grayish‐white when the CR‐stained section was observed under the fluorescent microscope with a 340–390 nm UV excitation light (Figure 2). The amyloid observed using these five methods showed consistency in tissue localization.

### 3.2 Diagnostic performance of the five methods

Among 82 PCA patients, amyloid was observed by CR‐UFM in 67 (81.71%) cases. The positive rate of other four methods were, respectively, 58 (70.73%) by CR‐PLM, 46 (56.10%) by CR staining, 25 (30.49%) by CV staining and 23 (28.05%) by HE staining. Statistical analysis indicated that the positive rate of CR‐UFM was significantly higher than that of CR‐PLM (*χ* ^2^ = 7.11, *p* = 0.004), CR staining (*χ* ^2^ = 19.05, *p* < 0.001), CV staining (*χ* ^2^ = 40.02, *p* < 0.001), or HE staining (*χ* ^2^ = 42.02, *p* < 0.001) (Table 1). Additionally, the mean relative number of positive dermal papillae observed by CR‐UFM (0.35 ± 0.27) was much more than that by CR‐PLM (0.15 ± 0.17, *Z* = −7.009, *p*<0.001), CR staining (0.12 ± 0.16, *z* = −6.955, *p* < 0.001), CV staining (0.07 ± 0.12, −7.115, *p* < 0.001), or HE staining (0.05 ± 0.12, *Z* = −7.009, *p* < 0.001) (Table 2).

**TABLE 1:** Comparison of positive rates in the diagnosis of PCA between CR‐UFM and the other four methods.

Results	Diagnostic methods				
	HE^a^	CV^b^	CR^c^	CR‐PLM^d^	CR‐UFM
Positive *n* (%)	23 (28.05)	25 (30.49)	46 (56.10)	58 (70.73)	67 (81.71)
Negative *n* (%)	59 (71.95)	57 (69.51)	36 (43.90)	24 (29.27)	15 (18.29)
*χ* ^2^ value	42.02	40.02	19.05	7.11	
*p* value	<0.001	<0.001	<0.001	0.004	

Abbreviations: CR, Congo red; CR‐PLM, Congo red under a polarized light microscope; CR‐UFM, Congo red ultraviolet‐emitted fluorescence microscope; CV crystal violet; HE, hematoxylin–eosin; PCA, primary cutaneous amyloidosis.

^a^ Comparison between HE and CR‐UFM.

^b^ Comparison between CV and CR‐UFM.

^c^ Comparison between CR and CR‐UFM.

^d^ Comparison between CR‐PLM and CR‐UFM.

**TABLE 2:** The comparison of relative number of positive dermal papillae between CR‐UFM and the other four methods.

Methods	Mean ± standard deviation		*Z* value	*p* value
	Pair 1	Pair 2		
HE/CR‐UFM	0.05 ± 0.12	0.35 ± 0.27	−7.009	<0.001
CV/CR‐UFM	0.07 ± 0.12	0.35 ± 0.27	−7.115	<0.001
CR/CR‐UFM	0.12 ± 0.16	0.35 ± 0.27	−6.955	<0.001
CR‐PLM/CR‐UFM	0.15 ± 0.17	0.35 ± 0.27	−7.009	<0.001

Abbreviations: CR, Congo red; CR‐PLM, Congo red under a polarized light microscope; CR‐UFM, Congo red ultraviolet‐emitted fluorescence microscope; CV crystal violet; HE, hematoxylin–eosin.

**FIGURE 3:**
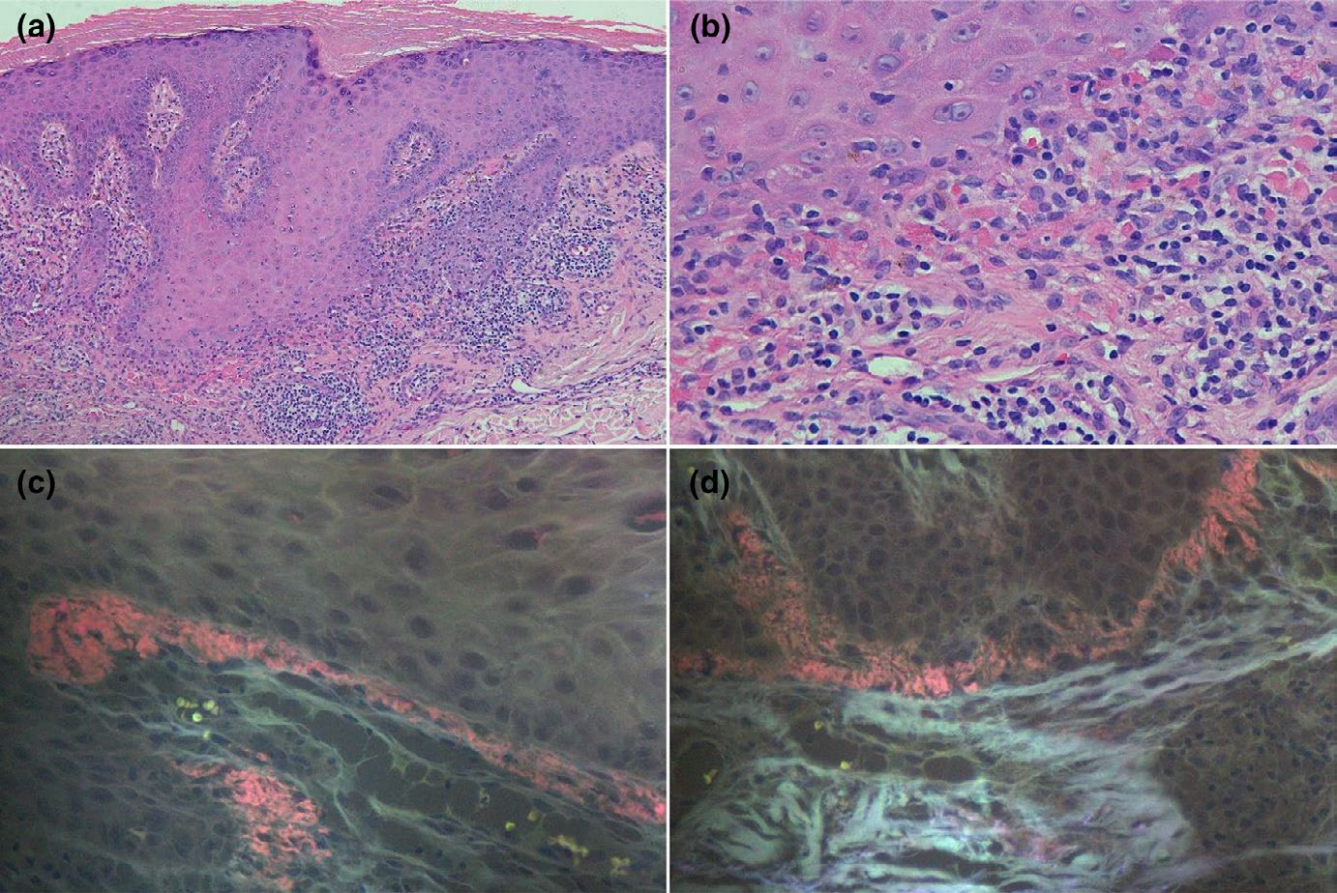
The deposits of bright purplish‐red ore‐like amyloid material amyloids were observed in the dermal papillae by CR‐UFM in one patient with lichen planus (original magnification a×100, b×200, c‐d×400).

In the control group, only one patient with lichen planus showed the presence of amyloid by CR‐UFM, the other four methods were negative. (Figure 3).

### 3.3 Comparison of CR‐PLM and CR‐UFM on the detection of amyloid

**FIGURE 4:**
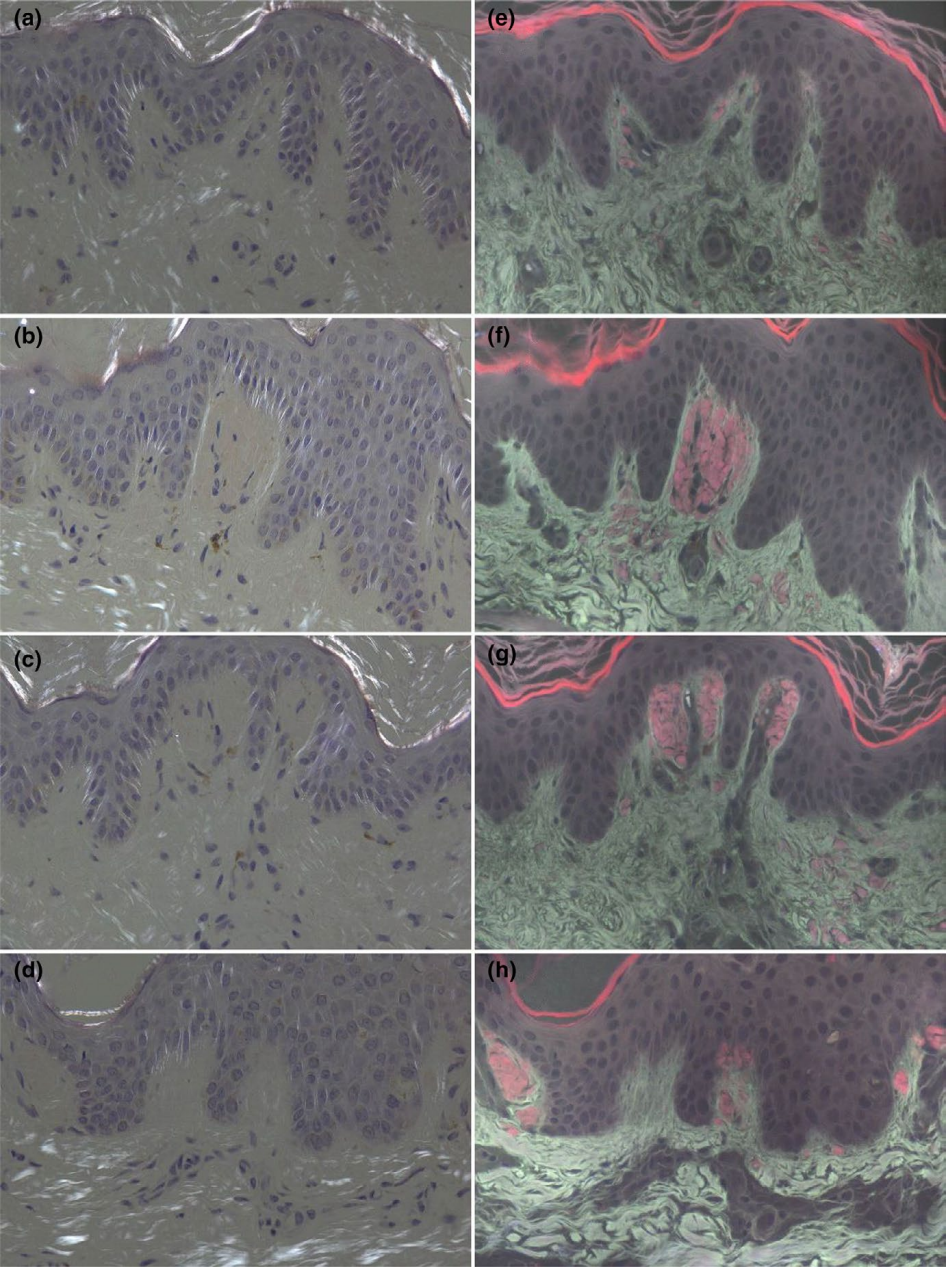
Some bright purplish‐red ore‐like amyloid material were observed by Congo red ultraviolet‐emitted fluorescence microscope (e–h), while negative observed by Congo red under a polarized light microscope (a–d) in the four primary cutaneous amyloidosis patients (original magnification×200).

**FIGURE 5:**
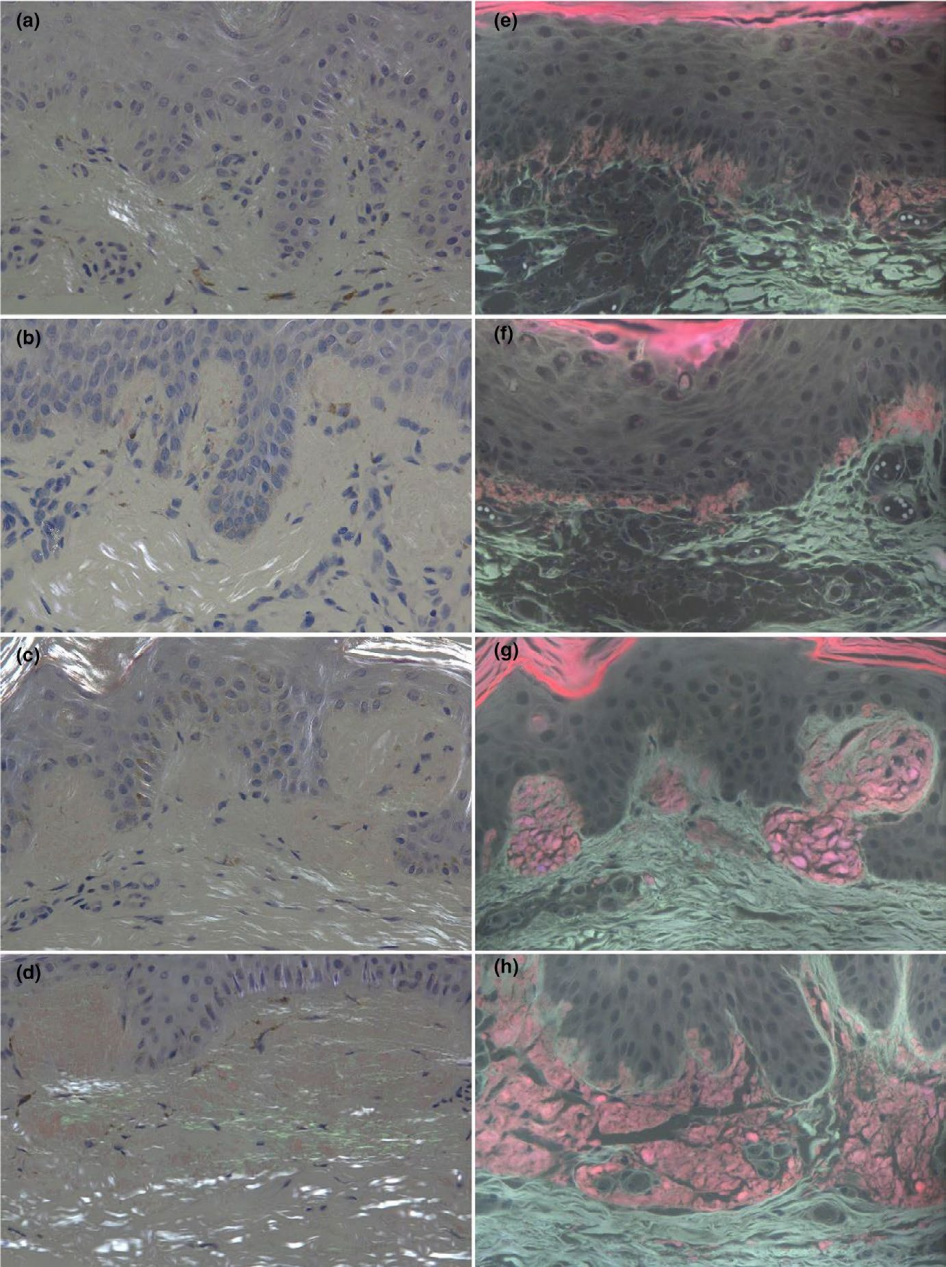
(a‐d) The grades of brightness intensity on the birefringence from Congo red under a polarized light microscope or fluorescence from an ultraviolet‐emitted fluorescence microscope represent none, weak, moderate and strong apple‐green birefringence respectively, (e–h) represent none, weak, moderate and strong fluorescence respectively (original magnification×200).

The purplish‐red ore‐like amyloid material were detected by CR‐UFM in nine patients who showed negative amyloid under CR‐PLM (Figure 4). Among 82 patients, 16 (19.51%) appeared as strong birefringence, 17 (20.73%) as moderate birefringence, 25 (30.49%) as weak birefringence, and 24 (29.27%) cases as no birefringence under CR‐PLM. Most of the apple‐green birefringence patterns were a short, curved line or dot‐like; a lump‐like pattern was rare. Sixty (73.17%) cases appeared as strong fluorescence, seven (8.54%) as moderate fluorescence, and 15 (18.29%) cases as no fluorescence under CR‐UFM (Figure 5). The brightness of fluorescence for CR‐UFM was significantly greater than that of apple‐green birefringence for CR‐PLM (*Z* = −6.440, *p* < 0.001) (Table 3).

**TABLE 3:** The comparison of the apple‐green birefringence brightness intensities observed by CR‐PLM with the fluorescence brightness intensities observed by CR‐UFM.

Results	Grade				*Z* value	*p* value
	0	1	2	3		
CR‐PLM *n* (%)	24 (29.27)	25 (30.49)	17 (20.73)	16 (19.51)	−6.440	<0.001
CR‐UFM *n* (%)	15 (18.29)	0 (0.00)	7 (8.54)	60 (73.17)		

*Note*: 0, 1, 2 and 3 represents none, weak, moderate and strong birefringence or fluorescence, respectively.

Abbreviations: CR‐PLM, Congo red under a polarized light microscope; CR‐UFM, Congo red ultraviolet‐emitted fluorescence microscope.

### 3.4 Amyloid deposits in 19 cases of systemic amyloidosis

**FIGURE 6:**
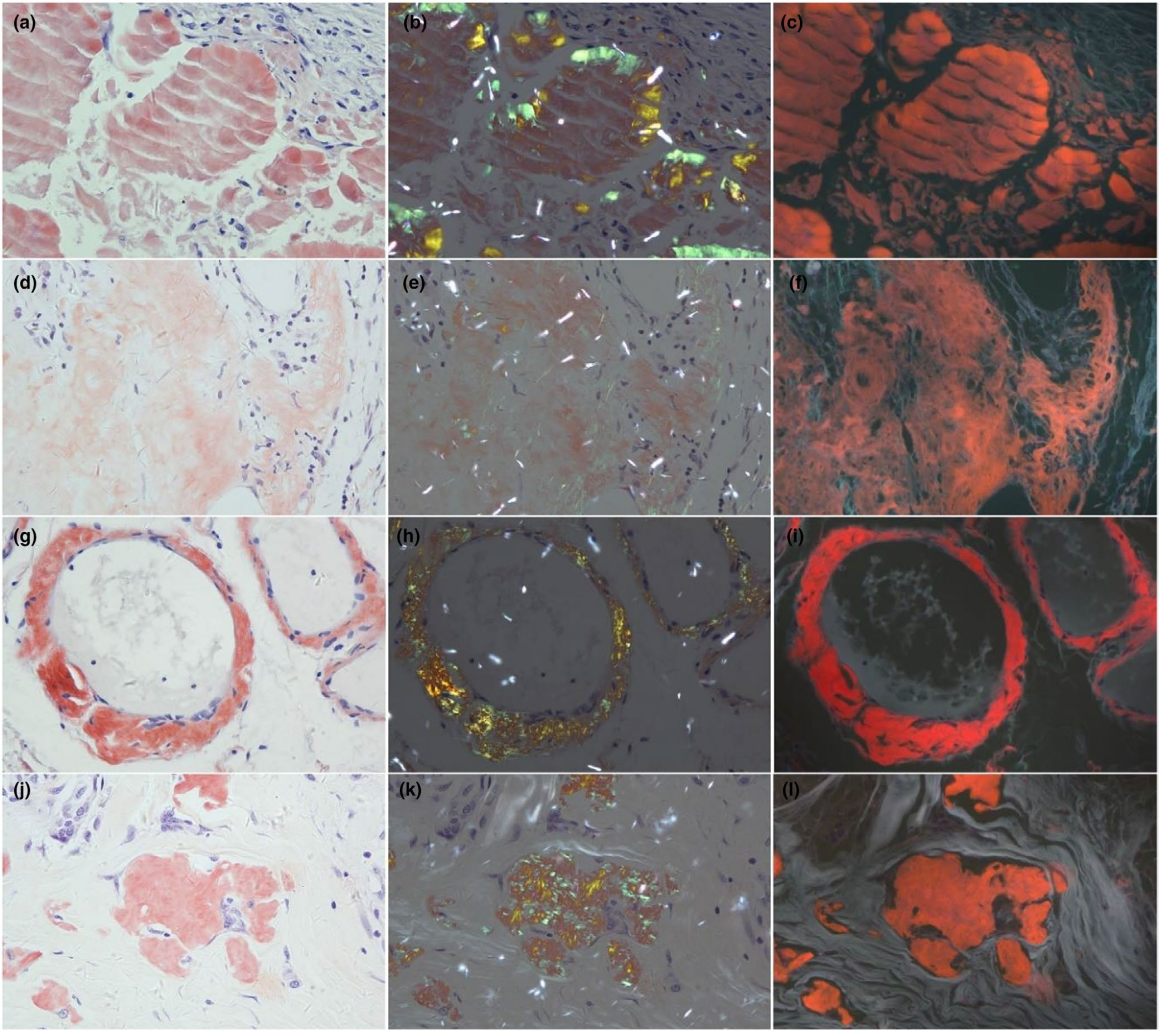
The amyloid protein under Congo red CR, Congo red under a polarized light microscope (CR‐PLM) and an ultraviolet‐emitted fluorescence microscope (CR‐UFM) of the lung (a–c), ureter (d–f), abdominal fat (g–i), and thyroid (j–l) tissue (original magnification×200).

The CR‐stained amyloid displayed as prominent brick‐red masses against the grayish‐white background of tissues under UFM in 19 cases of systemic amyloidosis (Figure 6).

## 4 DISCUSSION

Primary cutaneous amyloidosis (PCA) is a persistent pruritic dermatosis that primarily affects middle‐aged and elderly people.^3^ It is seen most frequently in China and Southeast Asia.^2^ Clinically, it is classified into three principal categories including macular amyloidosis (MA), lichen amyloidosis (LA), and nodular amyloidosis (NA).^21^, ^22^ The coexistence of macular and lichenoid lesions is known as biphasic amyloidosis (BA).^23^ Other rare types include anosacral amyloidosis, amyloidosis cutis dyschromica, and bullous amyloidosis.^24^, ^25^, ^26^ The current gold standard for diagnosing PCA is the apple‐green birefringence of CR‐stained amyloid deposits under a CR‐PLM.^27^ However, the apple‐green birefringence is not easily observed using CR‐PLM for most PCA patients. Therefore, it is necessary to develop a new diagnostic method.

In this study, we evaluated the application of five different methods in detecting amyloid deposits for the diagnosis of PCA, including HE, CV, CR staining, CR‐PLM, and CR staining under UV‐emitted fluorescence (CR‐UFM). The HE, CV and CR‐stained amyloid deposits showed as pink, purplish‐violet and brick‐red homogeneous amorphous material in the dermis under an ordinary light source, and were difficult to differentiate from the surrounding fibrous tissues. The CR‐stained amyloid deposits displayed the apple‐green birefringence of different brightness under a polarized light microscope, mostly a short, curved line or dot‐like patterns and, rarely, lump‐like patterns. Under UV fluorescence microscope (excitation filter 340–390 nm and absorption filter 420 IF), CR‐stained amyloid deposits appeared as bright purplish‐red ore‐like substances in the papillae of the dermis, while the color of the epidermal keratinocytes was gray, and the color of dermal tissues was grayish‐white. The amyloid deposits were more easily identified by CR‐UFM than with other four methods.

Among 82 patients with a clinical diagnosis of PCA, the positive rate of CR‐UFM was significantly higher than the other four methods. Moreover, it was also most accurate in detecting the relative number of positive dermal papillae. The fluorescence brightness intensities of CR‐UFM were significantly greater than the apple‐green birefringence brightness intensities of CR‐PLM. These findings suggest that the CR‐UFM is an excellent method to identify amyloid.

Small amounts of amyloid deposits observed by polarized light microscopy may lead to false‐negative results.^28^, ^29^ The Nomenclature Committee of the International Society of Amyloidosis highlighted that it was common to see red, green, yellow, and even mixed colors of amyloid protein in 2020.^30^ The colors of CR‐stained amyloid achieved by polarized light depend on the rotation degree of one filter compared with the other. A widespread insistence that amyloid protein shows “apple‐green birefringence” may be valid only if the polarizer and analyzer intersect precisely.^14^, ^16^ This intersection poses difficulties and challenges in operating microscopes and identifying amyloid.

Ultraviolet‐emitted fluorescence microscopy enhances the sensitivity in detecting CR‐stained amyloid, and its mechanism may be related to the specific chemical binding of CR dye to the amyloid and fluorescence property of CR dye. As an anionic azo dye, CR bonds primarily to β‐pleated sheets of amyloid protein by hydrogen bonding.^31^ The dye molecules are located in amyloid with specific spatial orientation. CR dye is a fluorochrome, exhibiting red fluorescence upon UV irradiation. Early in 1959, Cohen et al.^32^ were the first to apply CR fluorescence for detecting amyloid in an amyloidosis rabbit model. In 1965, Puchtler and Sweat^33^ found that the bright‐red amyloid protein stood out clearly against the pale greenish gray surrounding tissue when a darkfield condenser was used in combination with the UV‐blue absorption filter.

In 2000, Linke^16^ examined amyloid in 28 tissue samples of amyloidosis using Congo red fluorescence (CRF), the combination of CR staining and immunocytochemistry, and CR staining. In this study, CRF was confirmed to be the most sensitive detection method. The CR‐stained amyloid was illuminated in bright yellow‐orange with a dark green background using the fluorescein isothiocyanate (FITC) filter set (absorption maximum of 495 nm/emission maximum of 525 nm), and the CR‐stained amyloid protein was illuminated in bright red with a red background of lesser intensity using the tetrame thylrhodamine isothiocyanate filter set (absorption maximum of 555 nm/emission maximum of 580 nm). In 2012, Marcus et al.^17^ employed CRF and CR‐PLM to detect amyloid in 78 bone marrow specimens. CRF achieved 100% sensitivity, while the sensitivity of CRPLM was only 75%. The CR‐stained amyloid was illuminated in red‐orange with a dark green background under the excitation filter of FITC. In a recent study, Shehabeldin et al.^34^ examined amyloid in 92 tissue samples through Texas Red‐filtered fluorescence microscopy (TRFM). These samples were derived from the kidney, bone marrow, heart, fat, and maxillary sinus. The CR‐stained amyloid protein was illuminated in bright red with a red background of lesser intensity using the Texas Red filter (excitation peak 596 nm/emission maximum of 620 nm). TRFM increased the diagnostic yield and specificity of CR‐stained tissue sections by enhancing the congophilic areas. Fernandez‐Flores^35^ examined 12 cases of cutaneous amyloidosis and found that the positive rate of CR staining, thioflavin T staining, or CRF was 87.50%, while it was 100% for the immunohistochemical staining. The CR‐stained amyloid was illuminated in red with a dark background, but the fluorescence was not as strong as expected.

In our study, the excitation wavelength of the fluorescent microscope was set from 340 to 390 nm and the emission wavelength was set at 420 IF. Amyloid protein presents a bright purplish‐red or brick‐red ore‐like appearance which forms a sharp contrast with the surrounding grayish‐white dermal tissues or other organ tissues.

## 5 CONCLUSION

In conclusion, our study suggests that CR‐UFM is a sensitive method to identify the amyloid deposits, and worth popularizing and applying in the diagnosis of PCA. More studies are needed to further verify the diagnostic accuracy of this technique.

## FUNDING INFORMATION

This work was supported by the Natural Science Foundation of Universities of Anhui Province (2023AH053325).

## CONFLICT OF INTEREST STATEMENT

The authors declare no conflicts of interest.

## ETHICS STATEMENT

All procedures performed in studies involving human participants were in accordance with the ethical standards of the institutional and/or national research committee and with the 1964 Declaration of Helsinki and its later amendments or comparable ethical standards.

## Data Availability

The data that support the findings of this study are available from the corresponding author upon reasonable request.
